# Supplementary material for: Infective endocarditis post-transcatheter aortic valve implantation (TAVI), microbiological profile and clinical outcomes: A systematic review
Source: PLoS One. 2020 Jan 17;15(1):e0225077. doi: 10.1371/journal.pone.0225077 (PMC6968844; doi:10.1371/journal.pone.0225077)
Supplement: S1 Annexure — (DOCX) [file pone.0225077.s001.docx]

**S1 annexure: Search strategy**

| ((Transcatheter aortic valve implantation OR Transcatheter aortic valve replacement OR TAVI OR TAVR))) AND ((Endocarditis OR Infective endocarditis OR Prosthetic valve endocarditis))) AND ((Infective endocarditis after TAVI OR Incidence and clinical impact of infective endocarditis on TAVI OR TAVI-associated infective endocarditis OR Prosthetic valve endocarditis after transcatheter valve replacement OR Causative organisms of post-TAVI infective endocarditis OR Clinical outcomes of infective endocarditis after TAVI OR In-hospital mortality OR Mortality at follow-up OR Transcatheter heart failure OR Outcomes of TAVI)) |
| --- |
