# Supplementary material for: Infective endocarditis post-transcatheter aortic valve implantation (TAVI), microbiological profile and clinical outcomes: A systematic review
Source: PLoS One. 2020 Jan 17;15(1):e0225077. doi: 10.1371/journal.pone.0225077 (PMC6968844; doi:10.1371/journal.pone.0225077)
Supplement: S2 Annexure — (DOCX) [file pone.0225077.s002.docx]

**S2 annexure: Search strategy on Pubmed**

((Transcatheter aortic valve implantation OR Transcatheter aortic valve replacement OR TAVI OR TAVR))) AND ((Endocarditis OR Infective endocarditis OR Prosthetic valve endocarditis))) AND ((Infective endocarditis after TAVI OR Incidence and clinical impact of infective endocarditis on TAVI OR TAVI-associated infective endocarditis OR Prosthetic valve endocarditis after transcatheter valve replacement OR Causative organisms of post-TAVI infective endocarditis OR Clinical outcomes of infective endocarditis after TAVI OR In-hospital mortality OR Mortality at follow-up OR Transcatheter heart failure OR Outcomes of TAVI))


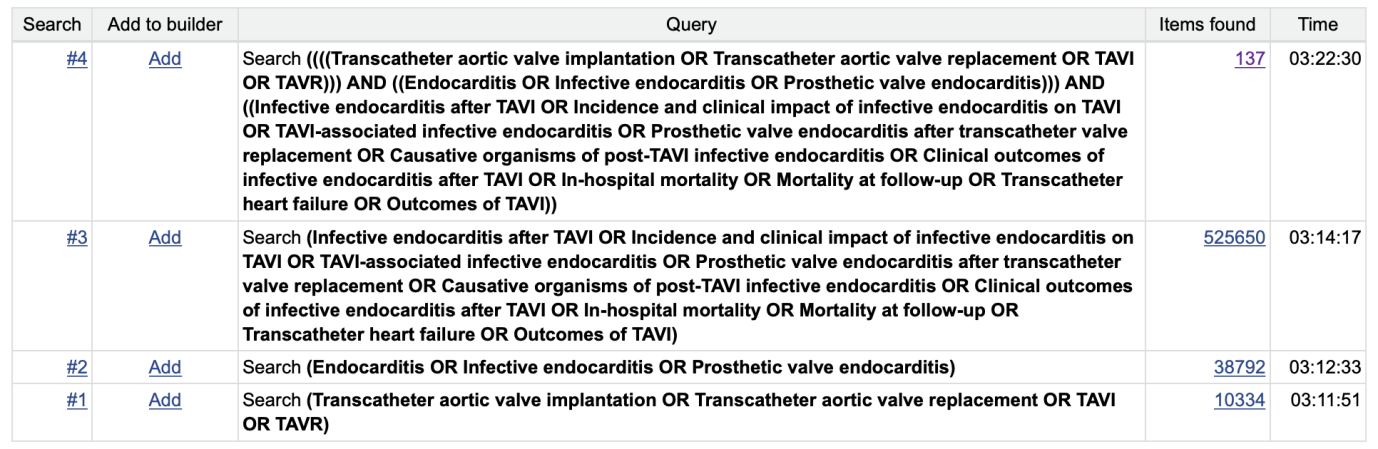


((("transcatheter aortic valve replacement"[MeSH Terms] OR ("transcatheter"[All Fields] AND "aortic"[All Fields] AND "valve"[All Fields] AND "replacement"[All Fields]) OR "transcatheter aortic valve replacement"[All Fields] OR ("transcatheter"[All Fields] AND "aortic"[All Fields] AND "valve"[All Fields] AND "implantation"[All Fields]) OR "transcatheter aortic valve implantation"[All Fields]) OR ("transcatheter aortic valve replacement"[MeSH Terms] OR ("transcatheter"[All Fields] AND "aortic"[All Fields] AND "valve"[All Fields] AND "replacement"[All Fields]) OR "transcatheter aortic valve replacement"[All Fields]) OR TAVI[All Fields] OR TAVR[All Fields]) AND (("endocarditis"[MeSH Terms] OR "endocarditis"[All Fields]) OR ("endocarditis"[MeSH Terms] OR "endocarditis"[All Fields] OR ("infective"[All Fields] AND "endocarditis"[All Fields]) OR "infective endocarditis"[All Fields]) OR (Prosthetic[All Fields] AND valve[All Fields] AND ("endocarditis"[MeSH Terms] OR "endocarditis"[All Fields])))) AND ((("endocarditis"[MeSH Terms] OR "endocarditis"[All Fields] OR ("infective"[All Fields] AND "endocarditis"[All Fields]) OR "infective endocarditis"[All Fields]) AND after[All Fields] AND TAVI[All Fields]) OR (("epidemiology"[Subheading] OR "epidemiology"[All Fields] OR "incidence"[All Fields] OR "incidence"[MeSH Terms]) AND clinical[All Fields] AND ("Impact (Am Coll Physicians)"[Journal] OR "impact"[All Fields]) AND ("endocarditis"[MeSH Terms] OR "endocarditis"[All Fields] OR ("infective"[All Fields] AND "endocarditis"[All Fields]) OR "infective endocarditis"[All Fields]) AND TAVI[All Fields]) OR (TAVI-associated[All Fields] AND ("endocarditis"[MeSH Terms] OR "endocarditis"[All Fields] OR ("infective"[All Fields] AND "endocarditis"[All Fields]) OR "infective endocarditis"[All Fields])) OR (Prosthetic[All Fields] AND valve[All Fields] AND ("endocarditis"[MeSH Terms] OR "endocarditis"[All Fields]) AND after[All Fields] AND transcatheter[All Fields] AND valve[All Fields] AND ("replantation"[MeSH Terms] OR "replantation"[All Fields] OR "replacement"[All Fields])) OR (Causative[All Fields] AND organisms[All Fields] AND post-TAVI[All Fields] AND ("endocarditis"[MeSH Terms] OR "endocarditis"[All Fields] OR ("infective"[All Fields] AND "endocarditis"[All Fields]) OR "infective endocarditis"[All Fields])) OR (Clinical[All Fields] AND outcomes[All Fields] AND ("endocarditis"[MeSH Terms] OR "endocarditis"[All Fields] OR ("infective"[All Fields] AND "endocarditis"[All Fields]) OR "infective endocarditis"[All Fields]) AND after[All Fields] AND TAVI[All Fields]) OR ("hospital mortality"[MeSH Terms] OR ("hospital"[All Fields] AND "mortality"[All Fields]) OR "hospital mortality"[All Fields] OR ("hospital"[All Fields] AND "mortality"[All Fields]) OR "in hospital mortality"[All Fields]) OR (("mortality"[Subheading] OR "mortality"[All Fields] OR "mortality"[MeSH Terms]) AND follow-up[All Fields]) OR (Transcatheter[All Fields] AND ("heart failure"[MeSH Terms] OR ("heart"[All Fields] AND "failure"[All Fields]) OR "heart failure"[All Fields])) OR (Outcomes[All Fields] AND TAVI[All Fields]))

Recent queries in pubmed

Search,Query,Itemsfound,Time

#4,"Search ((((Transcatheter aortic valve implantation OR Transcatheter aortic valve replacement OR TAVI OR TAVR))) AND ((Endocarditis OR Infective endocarditis OR Prosthetic valve endocarditis))) AND ((Infective endocarditis after TAVI OR Incidence and clinical impact of infective endocarditis on TAVI OR TAVI-associated infective endocarditis OR Prosthetic valve endocarditis after transcatheter valve replacement OR Causative organisms of post-TAVI infective endocarditis OR Clinical outcomes of infective endocarditis after TAVI OR In-hospital mortality OR Mortality at follow-up OR Transcatheter heart failure OR Outcomes of TAVI))",137,03:39:37

#3,"Search (Infective endocarditis after TAVI OR Incidence and clinical impact of infective endocarditis on TAVI OR TAVI-associated infective endocarditis OR Prosthetic valve endocarditis after transcatheter valve replacement OR Causative organisms of post-TAVI infective endocarditis OR Clinical outcomes of infective endocarditis after TAVI OR In-hospital mortality OR Mortality at follow-up OR Transcatheter heart failure OR Outcomes of TAVI)",525650,03:14:17

#2,"Search (Endocarditis OR Infective endocarditis OR Prosthetic valve endocarditis)",38792,03:12:33

#1,"Search (Transcatheter aortic valve implantation OR Transcatheter aortic valve replacement OR TAVI OR TAVR)",10334,03:11:51
