# Supplementary material for: Infective endocarditis post-transcatheter aortic valve implantation (TAVI), microbiological profile and clinical outcomes: A systematic review
Source: PLoS One. 2020 Jan 17;15(1):e0225077. doi: 10.1371/journal.pone.0225077 (PMC6968844; doi:10.1371/journal.pone.0225077)
Supplement: Attachment — Submitted filename: Response to Reviewers.docx [file pone.0225077.s004.docx]

The manuscript has been revised as suggested by the reviewers. Each point has been explained in this document. The marked and unmarked copies of the revised manuscript have been submitted.

**Journal Requirements:**

- The manuscript meets PLOS ONE's style requirements, including those for file naming.
- The Supporting Information files have been included at the end of manuscript.
- The dates during which the electronic search was performed have been mentioned.
- Table 2 has been omitted from the manuscript.
- The caption for figure 3 has been included.

**Answers to the Questions**

Reviewer's Responses to Questions

**Comments to the Author**

**4. Is the manuscript technically sound, and do the data support the conclusions?**
The manuscript must describe a technically sound piece of scientific research with data that supports the conclusions. Experiments must have been conducted rigorously, with appropriate controls, replication, and sample sizes. The conclusions must be drawn appropriately based on the data presented. 

Reviewer #1: Yes

Reviewer #2: Partly

Reviewer #3: Partly

Reviewer #4: Yes

The manuscript is technically sound and the data support the conclusions. The study is a systematic review which included all the relevant articles on post-TAVI infective endocarditis till October 2018. The literature search was systematically conducted on 3 electronic databases. Medline (Pubmed), Embase and the Cochrane Central Register of Controlled Trials. The conclusion of the study correlates with the data and is drawn from the results of the data analyzed.

**2. Has the statistical analysis been performed appropriately and rigorously?** 

Reviewer #1: Yes

Reviewer #2: No

Reviewer #3: N/A

Reviewer #4: N/A

The data is analyzed using mean, frequency & percentages and presented as tables & bar graphs. The meta-analysis could not be done as there was no comparison/control group.

**3. Have the authors made all data underlying the findings in their manuscript fully available?**

The PLOS Data policy requires authors to make all data underlying the findings described in their manuscript fully available without restriction, with rare exception (please refer to the Data Availability Statement in the manuscript PDF file). The data should be provided as part of the manuscript or its supporting information, or deposited to a public repository. For example, in addition to summary statistics, the data points behind means, medians and variance measures should be available. If there are restrictions on publicly sharing data—e.g. participant privacy or use of data from a third party—those must be specified.

Reviewer #1: Yes

Reviewer #2: Yes

Reviewer #3: No

Reviewer #4: Yes

The data of the manuscript is provided in the main text and supporting information. The data of systematic search is shown in data synthesis & PRISMA flow diagram. The important parameters of included students are tabulated in the results. The search strategy, Pubmed syntax and PRISMA checklist are provided in supporting information.

**4. Is the manuscript presented in an intelligible fashion and written in standard English?**
PLOS ONE does not copyedit accepted manuscripts, so the language in submitted articles must be clear, correct, and unambiguous. Any typographical or grammatical errors should be corrected at revision, so please note any specific errors here.

Reviewer #1: Yes

Reviewer #2: No

Reviewer #3: Yes

Reviewer #4: Yes

The manuscript is written in standard English which is clear and correct. Any grammatical mistake has been corrected in the revised manuscript.

**5. Review Comments to the Author**
Please use the space provided to explain your answers to the questions above. You may also include additional comments for the author, including concerns about dual publication, research ethics, or publication ethics. (Please upload your review as an attachment if it exceeds 20,000 characters)

**Reviewer #1:**
**Major issues**

- (Page 9, line 276) The data synthesis has been improved. The studies considered and the criteria which did not respect for the inclusion in the meta-analysis are mentioned.
- The antibiotic therapy during post-transcatheter aortic valve implantation infective endocarditis is not specified because the data on antibiotic therapy was not available in the included studies.
- The meta-analysis could not be done as there was no control/comparison group in the included studies.

**Minor issues**

- Name of the bacteria:

The name of Viridians streptococci has been changed to Viridans streptococci

Staphylococcus species: Species is written in non-italic style

- (Page 3, line 82) The Reference number is 1.
- (Page 3, line 83) The line has been rephrased.
- (page 9, line 278) The redundant 0 after the full stop has been deleted.
- (Page 12, table 5) The sum of the percentages of the microorganisms are corrected to make 100% in the Amat-Santos, Latib, Gallouche studies.
- (Page 12, table 5) Table 5 has been split into 2 different tables (one with the incidence & the etiology and one with the outcomes).
- (Page 13, line 331-332) In the relevant article, congestive heart failure is the presenting feature of post-TAVI IE. That is why the sum of the percentages are not 100%. The details have been mentioned in the manuscript.
- (Page 13, line 335, and in other lines) Enterococcus has been changed to Enterococci.
- (Page 14, line 347) The spelling of Staphylococcus has been corrected.
- (Page 14, line 351-353) The sentence has been rephrased.

**Reviewer #2:**

- Rationale: The references of sentences have been mentioned in the rationale.
- Method: The analysis plan has been described.
- Results: The results are expressed as tables and presented as Box plots. The risk of Bias is calculated and shown in table 3.
- The figures have been uploaded in the format (TIF and PACE) as suggested by the journal.
- Discussion: The discussion has been amended with the principle findings of the study.
- Limitations of the findings: The limitations have been added.
- Abbreviations have been pre-defined prior to use.

**Reviewer #3:**

**Major:**

- The manuscript has been checked for English mistakes.
- Introduction: TAVI and IE have been explained first with further details of post-TAVI IE. The central purpose and explanation of what the study adds to the knowledge are clearly mentioned in the rationale of the study.
- This systematic review is conducted on post-TAVI IE. The reference of a systematic review by Amat-Santos et al. has been mentioned in the discussion. Amat-Santos et al. conducted a systematic review on infective endocarditis after transcatheter aortic as well as pulmonary valve replacement.
- It has been stated in the introduction of the manuscript that the data on post-TAVI IE is limited and most of the available studies are case reports & case series. The systematic review by Amat-Santos et al. has been explained in the discussion. Most of the included studies in this review were also case reports and case series. The study by Ando et al. is a meta-analysis that compared the incidence of infective endocarditis following TAVI versus surgical aortic valve replacement. The study is published in 2019 whereas the literature search for our systematic review was conducted till October 2018.

Amat-Santos IJ, Ribeiro HB, Urena M, Allende R, Houde C, Bedard E, et al. Prosthetic valve endocarditis after transcatheter valve replacement: a systematic review. JACC Cardiovasc Interv. 2015 Feb; 8(2):334–46.  doi: 10.1016/j.jcin.2014.09.013.

Ando T, Ashraf S, Villablanca PA, Telila TA,Takagi H, Grines CL, et al. Meta-analysis comparing the incidence of infective endocarditis following after transcatheter aortic valve implantation versus surgical aortic valve replacement. Am J Cardiol. 2019 Mar 1; 123(5):827-32. doi: 10.1016/j.amjcard.2018.11.031.

- The differences and similarities between native valve-IE and TAVI-IE; in particular, concentrating on different timing and diagnostic management have been mentioned in the introduction.
- The review does not improve the discussion on IE prophylaxis, although the Rationale sets this topic as a central objective. This is because the data on IE prophylaxis is not detailed in the included studies.
- The case reports and case series were excluded from this systematic review. This is because most of the included studies in the two systematic reviews (available in the literature) on post-TAVI IE were case reports & case series. The major limitation of these reviews was that it may have precluded the real evaluation of the post-TAVI infective endocarditis and certain cases of post-TAVI infective endocarditis might not have been published leading to potential publication bias. The details of these two systematic reviews by Amat-Santos et al. [26] & Eisen et al. [19] and their limitations have been mentioned in the discussion.
- The results of the study have been explained in the discussion along with comparison with the results of two systematic reviews available. The results of our systematic review cannot be compared with the findings of the included studies. The details of the included studies have been mentioned in the discussion.
- The studies by Onsea et. al., Buellesfeld et al. and Barbanti et al. were not included in the systematic review because these studies do not meet the inclusion criteria. The details of these studies with their references are given below:
- In a study by Onsea et al., a total of 73 TAVI patients were analyzed for the development of complications. Eleven patients developed infections but none of them was diagnosed with infective endocarditis [1].
- In a study by Barbanti et al., the patients who underwent TAVI were categorized into two groups: those with Society of Thoracic Surgeons (STS) score ≤7% and patients with a score >7%. The outcomes of TAVI were compared between these 2 groups. The results showed that the patients with STS ≤7% had lower rates of all-cause and cardiovascular mortality at 3 years after transcatheter aortic valve implantation.
- In another study by Barbanti et al., the outcomes of TAVI were compared in patients discharged within 72 hours and after 3 days of the procedure. The study showed that discharge within 72 hours after TAVI is feasible and does not seem to jeopardise the early safety of the procedure [3].
- Buellesfeld et al. evaluated the outcomes and predictors of success of procedure in patients who underwent TAVI at two institutions in German & Switzerland. The reported rates of in-hospital mortality, myocardial infarction and stroke were 11.9%, 1.8% and 3.6%, respectively.

1. Onsea K, Agostoni P, Voskuil M, Samim M, Stella PR. Infective complications after transcatheter aortic valve implantation: results from a single centre. Neth Heart J. 2012; 20:360–4. doi: 10.1007/s12471-012-0303-9.
2. [Barbanti](javascript:void(0);) M, [Schiltgen](javascript:void(0);) M, [Verdoliva](javascript:void(0);) S, [Bosmans](javascript:void(0);) J, [Bleiziffer](javascript:void(0);) S, [Gerckens](javascript:void(0);) U, et al. Three-year outcomes of transcatheter aortic valve implantation in patients with varying levels of surgical risk (from the CoreValve ADVANCE Study). Am J Cardiol. 2016; 117(5):820-7.
3. Barbanti M, Capranzano P, Ohno Y,  Attizzani GF, Gulino S,  Imme S, et al. Early discharge after transfemoral transcatheter aortic valve implantation. Heart. 2015; 101:1435-6. doi: 10.1136/heartjnl-2015-308415.
4. Buellesfeld L, Wenaweser P, Gerckens U, Mueller R, Sauren B, Latsios G, et al. Transcatheter aortic valve implantation: predictors of procedural success—the Siegburg–Bern experience. Eur Heart J. 2010; 31:984–91. doi:10.1093/eurheartj/ehp570.

- There were only two articles reporting on stroke after post-TAVI IE. The mean of stroke incidence may be influenced by extreme values (0% vs. 10.3%). The mean of stroke has been omitted from the manuscript.
- Infective endocarditis outcomes have not been integrated with the impact of different treatment options (i.e. antibiotics therapy, surgery). Because none of the included study correlated the outcomes with the treatment modality.

**Minor:**

- Scientific English and grammar have been improved.
- The green underscore has been omitted in figure 3.
- Corrected copy of figure 4 has been submitted.
- Corrected copy of figure 5 has been submitted.
- Redundant “0” in line 278 has been omitted.
- The sign of % has been added after valve-in-valve procedure” in Table 5. In addition, some words which appear in italic as a mistake are corrected.

**Reviewer #4:**

- The timeline of the literature search has been mentioned in the method section of the abstract.
- The first and second authors were the principal investigators who performed the literature search. It has been mentioned under the heading of data management, selection and data collection.
- Figure 3 (PRISMA flow diagram) has been made by using PRISMA 2009 Flow Diagram platform.
- The figures have been uploaded in the format suggested by the journal.
- The syntax used in Pubmed is attached in the supplementary file.
