# Supplementary material for: Infective endocarditis post-transcatheter aortic valve implantation (TAVI), microbiological profile and clinical outcomes: A systematic review
Source: PLoS One. 2020 Jan 17;15(1):e0225077. doi: 10.1371/journal.pone.0225077 (PMC6968844; doi:10.1371/journal.pone.0225077)
Supplement: Attachment — Submitted filename: Response to Reviewers - Copy.docx [file pone.0225077.s005.docx]

Reviewer 1:

The patients of post-TAVI IE were diagnosed according to the Modified Duke’s criteria. The data on antibiotic prophylaxis is detailed in 2 studies by Regueiro et al. and Amat-Santos et al. and it has been mentioned in the Results. The antibiotic therapy for post-TAVI IE has also been included in the manuscript. Recommendations have been added based on the conclusion of the manuscript.

Reviewer 3:

- The definition of sepsis and septic shock are included in the manuscript in study outcomes (Methodology).
- All abbreviations are first preceded by complete word. After that, only abbreviations are written in the manuscript.
- The grammatical mistakes of the line no. 418 (now line no. 457) have been corrected.

Reviewer 4:

The antibiotics used for treating post-TAVI IE are given in 5 studies. These details have been added in the results. The patients undergoing surgical treatment (valve explantation and valve-in-valve procedure) have already been included in the results. Due to the heterogeneity of data in the included studies, further analysis of the variables cannot be done.
